# A prospective study into the incidence of aspiration and dysphagia in acute stroke patients admitted to Beaumont Hospital

**DOI:** 10.1186/1753-6561-9-S7-A13

**Published:** 2015-10-27

**Authors:** Tristam Hills, Michael Walsh

**Affiliations:** 1 Royal College of Surgeons in Ireland, Dublin, Ireland; 2 Ear, Nose, Throat Department, Beaumont Hospital, Dublin, Ireland

## Introduction

Stroke is the second leading cause of death in the world [1]; 1.3-5% of these deaths are caused by aspiration pneumonia [2]. Dysphagia occurs in 50% of stroke cases; 60% of these cases can lead to silent aspiration [3]. This study aims to assess the incidence of aspiration and dysphagia in the Beaumont Hospital Acute Stroke Unit (BHASU), while discussing the use of the Beaumont Hospital Swallow Screen (BHSS) and subsequent investigations, involving fiberoptic endoscopic evaluations of swallowing (FEEs) and videofluoroscopy.

## Methods

The inclusion criteria were patients with acute stroke, referred to Beaumont Hospital between 1^st^ of April and 26^th^ of August 2013. Data was collected -both prospectively and retrospectively- from a systematic review of patient's charts, Beaumont Hospital Speech and Language Department records and the BHASU records. Data included swallow screen results, dysphagia diagnosis, aspiration events and records of subsequent investigations. Collected data was collated and analysed for incidence of aspiration, dysphagia, BHSS use and subsequent investigations.

## Results

97 patients met the inclusion criteria.

• 29 (30%) were diagnosed with dysphagia, 4 (4.1%) patients aspirated

• 61 (63%) patients were referred to Beaumont Speech and Language Department

• 43 (44%) received the BHSS, 54 (56%) did not

9 FEEs and 7 videofluoroscopy studies were carried out.

## Conclusions

Rates of aspiration and dysphagia are much lower than previous research would indicate. The BHSS is a sensitive and specific screening tool; however acute stroke patients who are not admitted directly to the BHASU are not being screened with the BHSS. Screening in the Accident and Emergency department as part of a stroke work-up would increase the number of patients receiving the BHSS. A permanent FEEs clinic would be of use in Beaumont Hospital for the prompt diagnosis or exclusion of suspected aspiration. The conclusions of this study are limited by the small sample size available.
